# Noug Seed (*Guizotia abyssinica*) Cake Substituted with Dried Mulberry (*Morus indica*) and *Vernonia amygdalina* Mixed Leaves' Meal on Growth Performances of Bonga Sheep at Teppi, Ethiopia

**DOI:** 10.1155/2020/9308761

**Published:** 2020-09-25

**Authors:** Gezahegn Mengistu, Getnet Assefa, Samuel Tilahun

**Affiliations:** ^1^Teppi Agricultural Research Center, P.O. Box: 34, Teppi, Ethiopia; ^2^Ethiopian Institute of Agricultural Research, P.O. Box: 2003, Addis Ababa, Ethiopia; ^3^Jimma University, College of Agriculture and Veterinary Medicine, P.O. Box: 307, Jimma, Ethiopia

## Abstract

This study was conducted to evaluate noug seed (*Guizotia abyssinica*) cake substituted with dried mulberry and *Vernonia* mixed leaves' meal on feed intake, body weight change, and digestibility of Bonga sheep fed on Rhodes grass hay at Teppi Agricultural Research Center, Ethiopia. The experiment was conducted using 25 yearling lambs with an average initial body weight of 20.83 ± 1.66 kg. Five lambs were allotted per treatment in randomized complete block design. Treatments were isonitrogenous supplements of 100% concentrate (49.5% NSC, 49.5% ground maize grain, and 1% salt) offered at 400 g DM/head/day (T1), 25, 50, 75, and 100 of NSC CP substituted by dried mulberry and *Vernonia* mixed leaves' meal in T2 = 429.8 g/day, T3 = 459.5 g/day, T4 = 489.3 g/day, and T5 = 519 g/day, respectively. The sheep were fed Rhodes grass hay *adlibitum* and had free access to water and salt block. Intake and growth trail lasted for 90 days followed by 10 days of digestibility trial. The CP, NDF, and ADF contents of Rhodes grass hay were 7.9, 74.8, and 46.8 percent. The CP content of NSC, ground maize grain, dried mulberry, and *Vernonia* leaves was 32.4, 8.3, 18.5, and 22.5%, respectively. Total DM intake (g/day) was significantly higher for lambs in T3 (771.4) than in T1 (722.8) and T5 (642.8) but similar to T2 (754.9) and T4 (759.7). The CP intake was highest (*P* < 0.001) for sheep in T2 and T3, but lowest in T5. The apparent DM digestibility for T1 (70.8%), T2 (68.8%), and T3 (69.1%) was significantly higher than for T5 (64.4%), while T4 (67.9%) and T5 were not significantly different. The attained average daily gain (g/day) of sheep in T1 (87.7), T2 (82.0), T3 (83.4), and T4 (75.2) was higher (*P* < 0.01) than in T5 (56.0). The result of this study indicated that dried mulberry and *Vernonia* mixed leaves' meal can substitute NSC as a protein supplement up to 75% inclusion level resulting in optimum DM and nutrient intakes and body weight gain of yearling Bonga sheep. This study also highlights the positive potential of dried mulberry and *Vernonia* mixed leaves' meal as a supplement to ruminants on a basal diet of fibrous feeds.

## 1. Introduction

Sheep are small ruminants capable of exploiting poor, unfertile, desert, and mountainous terrains, where other classes of livestock find difficulties in living and producing economically, Kumar et al. [1]. Ethiopia is one of the African countries with the largest small ruminant population in the continent [2]. The recent estimate indicates that there are about 30.7 million heads of sheep in the country, out of which 72.2 percent are females, and the rest are males [3]. Bonga sheep are one of the Ethiopian recognized breed types found in humid and mid-highland (1200–2500 meters) ecological zones and are geographically distributed in Keffa, Sheka, and Bench zones of Southern Nations, Nationalities, and Peoples' Regional State (SNNPRS) region [4, 5].

Lack of dietary nutrients, mainly energy and protein, is the major factor affecting productivity of sheep. Both energy and protein are equally important during the dry season for optimum weight gain [6]. Proteins are the major components of the animal body and are continuously needed in the feed for growth and cell repair [7]. However, the use of protein supplements is limited under smallholder livestock producers due to unavailability and high costs. Moreover, available feed resources are not properly and efficiently utilized by livestock producers, Ebro et al. [8].

In Southwestern Ethiopia, Keffa, Bench-Maji, and Sheka zones, smallholder farmers practiced traditional fattening of lambs during off-seasons. This enables farmers to generate income from the sale of live animals most commonly during holidays and also for their own home consumption. However, most farmers in the study area are constrained with lack of protein source supplements due to unavailability and high costs. In order to alleviate problems associated with protein supplements, there is a need to look for alternative protein source feeds which are preferably locally available and easily accessible by farmers. Thus, supplementation with indigenous and introduced multipurpose fodder trees and shrub leaves has great advantage. Among indigenous multipurpose tree and shrub fodders, *Vernonia amygdalina* are locally available, have considerable nutritional value, can be grown by farmers, and play a significant role as a protein supplement for ruminants, Aynalem and Taye [9]; Takele et al. [10]. Similarly, mulberry (*Morus indica)* is a browse tree with good ability to produce quality forage for livestock in humid and subhumid agroecologies [11]. Besides their easy accessibility, these foliage trees provide high-quality feed, particularly the protein content (15–28% CP), which could be an alternative replacement to conventional and expensive feed resources [12]. Although mulberry is introduced and widely utilized as a source of forage for silk worms in different parts of Ethiopia [13] including southwestern parts, so far no adequate study has been conducted and information is available for users regarding its role as sheep feed in the area. Therefore, this study was designed to evaluate substituting of noug seed cake of concentrate mix with dried mulberry and *Vernonia* mixed leaves' meal feed intake, digestibility, and growth performances of growing Bonga sheep and to identify the optimum substitution level of dried mulberry and *Vernonia* mixed leaves' meal fed on Rhodes grass hay as a basal diet.

## 2. Materials and Methods

### 2.1. Experimental Feed Preparation and Feeding

The experiment was undertaken in southwest Ethiopia at Teppi Agricultural Research Center (7^o^ 08′N; 35^o^ 18′ E). Rhodes grass (Massaba variety) was established at Teppi Agricultural Research Center. The grass was harvested at about 50% flowering stage manually using sickles and sun-dried in open air for three to four days until the moisture content of the hay was about 15% to 20%. The dried hay was piled and stored as loose hay under shade. The hay was manually chopped to a size of approximately 5-6 cm to facilitate intake.

The mulberry (*Morus indica)* and *Vernonia* (*Vernonia amygdalina)* leaves' meal was prepared from the trees grown in the research center. Green leaves of the plant were harvested by cutting with sickle manually from the branches during the months of October to November. The leaves from bottom to top of the branches were collected from the standing shrub of mulberry and *Vernonia* to make the feed collected to be representative. The harvested leaves were subjected to air-drying under shed separately for three to four days and turned up four times a day to ensure uniform drying and maintain green color. The dried mulberry and *Vernonia* leaves were crushed with hands by twisting and stored under shed until used for the feeding trial to maintain their quality.

Noug seed (*Guizotia abyssinica*) cake (NSC) was purchased from Addis Ababa oil processing factory. Maize grain was purchased from Teppi town and crushed/ground by the local grain miller and stored at the experimental site. To conduct the feeding trial, adequate amount of all the experimental feeds (Rhodes grass hay, *Vernonia* and mulberry leaf meal, and the concentrate mix) was prepared once before the commencement of the experiment. The grass hay was offered *ad libitum*, and the supplements were given twice daily in equal portions at 8:00 am and 16:00 pm to each experimental lamb [14].

### 2.2. Experimental Animals' Management

Twenty-five intact yearling Bonga lambs were used for the feeding trial. The lambs were purchased from the local market in Yeki district, Teppi town, Ethiopia. The lambs were purposively selected and purchased with similar ages, in which the ages were estimated by dentition. The experimental animals were ear-tagged, treated with albendazole (150 mg/head) for internal parasites, and vaccinated with ovine pasteurellosis and quarantined for 3 weeks before the start of the experiment. Then, the animals were housed in a well-ventilated individual pen consisting of a feeding trough for hay, separated plastic bucket for supplemental diets, and water troughs. The lambs were fed individually with the experimental diets for an adaptation period of 2 weeks followed by 90 days of feeding period and 10 days of digestibility trial. Water and salt block were available to all animals free access to all days during the feeding period. The pens and the troughs were cleaned everyday early morning before offering the daily experimental feeds.

### 2.3. Experimental Design and Treatment Layout

The feeding trial was arranged in a randomized complete block design (RCBD) containing 5 treatments and 5 replications. Initial body weight of lambs was applied as a block, and the experimental lambs were randomly assigned to the dietary treatments within the block. The average initial body weight of experimental lambs was 20.83 ± 1.66 kg (Mean ± SD). The proportion of feed ingredients of the concentrate mixture for the supplement in the control treatment was 49.5% NSC, 49.5% ground maize grain, and 1% common salt and set at 400 g/head/day based on the previous recommendation for finishing Horro lambs [15].

The dietary treatments containing inclusion of mixed leaves meal (T2, T3, T4, and T5) were prepared for NSC of concentrate mix at different levels. The proportion of the dried mulberry and *Vernonia* leaves' meal was calculated based on the CP content of NSC and mixed in one-to-one ratio for the substitute of NSC to make supplements isonitrogenous in all treatments.

The amount of supplements was fixed to supply 64.15 g CP which falls between 60 and 65 g CP/head/day which is recommended for growing sheep weighing 20–30 kg and gaining 100 g/head/day [16]. The dietary treatments were a replacement of the protein in noug seed cake of concentrate mix at 25, 50, 75, and 100% proportions with dried mulberry and *Vernonia* mixed leaves' meal. The amount of the supplements was 400.0, 429.8, 459.5, 489.3, and 519.0 g/day/head on DM basis for T1, T2, T3, T4, and T5, respectively. Rhodes grass hay was offered *ad libitum* (Table 1).

### 2.4. Measurements

#### 2.4.1. Feed Intake

The daily feed offered and the corresponding refusals of hay and supplemental diets were weighted and recorded daily for each experimental animal during the entire period of the experiment (90 days) to determine daily feed intake. The DM intake was calculated as the difference between amount of feed offered and refused on dry matter basis. Similarly, nutrient intake was computed as the difference between nutrient content of feed offered and nutrient content in feed refusals on DM basis. The estimated metabolisable energy (EME) intake of experimental animals was estimated from its digestible organic matter intake (DOMI) by using the following formula: EME (MJ/kg DM) = DOMI ×  0.0157 [17].

#### 2.4.2. Body Weight and Feed Conversion Efficiency

The live body weight of each animal was taken at every 10-day interval after depriving of the animals from feed and water for16 hours before each weighing [18]. The weight of sheep was taken using a personal digital balance by caring each sheep with known weight of an individual person and then subtracting the weight of the person from the total weight. The initial and final live weight of the experimental animals were used to determine the weight change during the 90-day feeding period. Average daily body weight gain was calculated as the difference between final and initial body weight divided by the number of feeding days. Feed conversion efficiency (FCE) is the measure of feed utilization how efficient the lambs were converting the feed into meat and calculated according to the following formula [19]:

(1) $$\begin{array}{c} \text{feed conversion efficiency}=\frac{\text{average daily live weight gain }\left(\text{g}\right)}{\text{average daily feed intake }\left(\text{g}\right)}. \end{array}$$

#### 2.4.3. Digestibility Trial

The digestibility trial of the treatments diets was conducted using all experimental animals at the end of the feeding trial for ten days. The experimental animals were allowed to adapt harnessing of faecal collection bags for three days before data collection. The actual data collection was done for seven days. The daily feed offered and refused and faecal outputs were measured for each animal based on DM basis. Dry matter of the faecal output was determined daily. Representative faecal samples (10% from total faecal production) were dried at 65°C for 72 hours to constant weight in a forced draft oven for dry matter determination. At the end of the collection period, the subsamples of partially dried faeces were pooled from each animal, and 10% subsample was ground to pass through a 1 mm sieve and stored in an airtight polyethylene bag pending analysis. The chemical analysis was undertaken at the animal nutrition laboratory of Holeta Agricultural Research Center. Apparent digestibility of DM, OM, CP, NDF, and ADF was determined as a percentage of the nutrient intake not recovered in the faeces using the following equation [20]:

(2) $$\begin{array}{c} \text{apparent nutrient digestibility }\left(\text{\%}\right)\;=\;\frac{\text{nutrient intake}-\text{nutrient in feaces}}{\text{nutrient intake}}*100\text{.} \end{array}$$

#### 2.4.4. Chemical Analysis of Feed and Faeces

The feed samples used in feeding (offered and refusal) and faecal samples were analyzed for their chemical composition at Holeta Agriculture Research Center. The offered samples were analyzed before the start of the feeding trial to be used in the ration formulation. Samples of feed offered were collected from each treatment, while samples of refusal were taken from each sheep daily. Collected samples were pooled per treatment over the experimental period and stored in plastic bags pending analysis.

The subsamples of feed offered and refusal were ground to pass through a 1 mm sieve screen and were ready for chemical analysis. Offered and refusal samples of feed were dried at 105°C overnight in a forced draft oven for DM determination, while the ash content was determined by burning/igniting feed samples in a muffle furnace at 550°C. The DM, ash, and organic matter contents of the feed samples were determined following the procedure of AOAC [21]. The N content of the samples was determined according to the micro-Kjeldahl method [21], and the crude protein (CP) was calculated as N∗6.25. Neutral detergent fiber (NDF), acid detergent fiber (ADF), and acid detergent lignin (ADL) were determined according to the procedure of Van Soest and Robertson [22].

### 2.5. Data Analysis

The data obtained on feed and nutrient intakes, digestibility, and body weight change were subjected to analysis of variance (ANOVA) using the general linear model procedures of the statistical analysis system [23]. Duncan's multiple range tests were used for mean separation that was found to be statistically different at the 5% significant level. The statistical model used for the analysis of data is

(3) $$\begin{array}{c} Y_{ij}=\mu +\;T_{i}+B_{j}+E_{ij}, \end{array}$$

where *Y*_*ij*_ = response variable, *μ* = overall mean, *T*_*i*_ = treatment effect, *B*_*i*_ = block effect, and *E*_*ij*_ = random error.

## 3. Results

### 3.1. Chemical Analysis of Experimental Feeds and Treatments

The chemical compositions of the experimental feed ingredients and treatment diets used in the present study are tabulated in Table 2. The Rhodes grass hay used as a basal diet in the current study was poor in quality. The crude protein content of the refusal hay was lower compared to the offered grass hay. The CP concentrations of the dried mulberry leaf meal (DMLM) and dried *Vernonia* leaf meal (DVLM) were lower than noug seed cake. Mulberry leaf meal had lower NDF than *Vernonia* leaf meal, whereas both had similar ADF.

### 3.2. Feed Intake

The mean daily DM intake of feeds, total DM, and nutrient intakes of growing Bonga lambs during 90 days of an experimental period are presented in Table 3. The lambs allotted in treatments T1 and T2 consumed all quantities of supplement offered without refusal during the entire experimental period. Significant differences (*P* < 0.01) were observed among treatments in daily hay DM and total DM intakes. Intake of Rhodes grass hay dry matter was significantly different (*P* < 0.01) among treatments. The basal diet intake for lambs fed in T2 (325.9 g/day) observed the highest value, whereas T5 showed the lowest value (228.1 g/day). The statistically differences (*P* < 0.01) of basal diet intake of lambs were described as T1 = T3 = T2 > T4 > T5. Total dry matter intake (TDMI) of sheep receiving T1, T2, T3, and T4 was significantly higher (*P* < 0.01) than T5. Total organic matter intake of sheep allotted in T5 was significantly lower than sheep assigned in the rest four treatments, and the trend was also similar with organic matter (OM) intake and estimated metabolisable (EME) intake. The CP intake of sheep grouped in the noug seed cake inclusion was significantly higher than sheep allotted in mulberry and *Vernonia* mixed leaf meal treatments (T5). Significantly lower (*P* < 0.001) ash and ADL intake were observed on sheep assigned in T1 than in T2, T3, T4, and T5. Higher NDF intake was recorded in sheep maintained on treatments containing different proportions of NSC than sheep fed T5.

**Figure 1:**
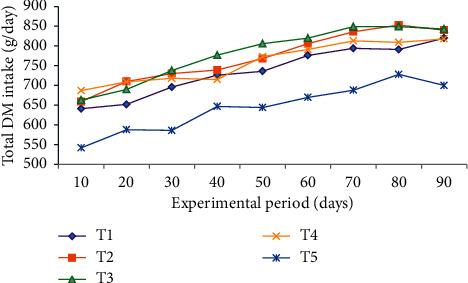
Trends of total DM intake during the experimental period of Bonga lambs fed on Rhodes grass hay basal diet and supplemented with dried mulberry and *Vernonia* mixed leaves' meal substituting noug seed cake of concentrate mix at different levels. T1 = Rhodes grass hay *ad libitum* + 100% concentrate mix; T2 = Rhodes grass hay *ad libitum* + 75% noug seed cake (NSC) and 25% dried mulberry and *Vernonia* mixed leaves' meal; T3 = Rhodes grass hay *ad libitum* + 50% NSC and 50% dried mulberry and *Vernonia* mixed leaves' meal; T4 = Rhodes grass hay *ad libitum* + 25% NSC and 75% dried mulberry and *Vernonia* mixed leaves' meal; T5 = Rhodes grass hay *ad libitum* + 100% dried mulberry and *Vernonia* mixed leaves' meal.

Trends of total dry matter intake (TDMI) over the experimental period are illustrated in Figure 1. Total dry matter intake increased with advancing feeding periods.

### 3.3. Apparent Dry Matter and Nutrient Digestibility

The apparent DM, OM, CP, NDF, and ADF digestibility percentage of experimental feeds are presented in Table 4. The apparent DM and OM digestibility percentages of T5 were significantly different (*P* < 0.05) and lower than the rest of treatments. The apparent CP digestibility percent value of T1 (81.8%) was the highest, whereas T5 (73.9%) showed the lowest. The digestibility of NDF was significantly (*P* < 0.001) different among the experimental diets where T1 showed the highest digestibility.

### 3.4. Body Weight and Feed Conversion Efficiency

Mean values of initial body weight, final body weight, body weight change, average daily gain, and feed and protein conversion efficiencies (FCE and PCE) of experimental animals are shown in Table 5. There were significant differences (*P* < 0.01) among treatments on body weight changes (BWC) and average daily weight gain (ADG). Thus, sheep in T1, T2, T3, and T4 had higher (*P* < 0.05) body weight changes than sheep received in T5, but T1, T2, T3, and T4 had no significant difference in terms of BWC and ADG. There was no significant variation observed in feed conversion efficiency (FCE) and protein conversion efficiency (PCE).

**Figure 2:**
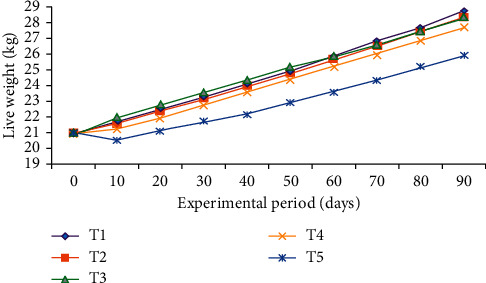
Trends in body weight changes of growing Bonga lambs fed on Rhodes grass hay basal diet and supplemented with dried mulberry and *Vernonia* mixed leaves' meal as a substitute to NSC of concentrate mix during the 90-day feeding period.

The trend of body weight change of experimental animals across the feeding period is shown in Figure 2. The body weight change over the 90 days of feeding period showed an increment trend.

### 3.5. Regression Analysis of Protein Intake and Daily Weight Gain

**Figure 3:**
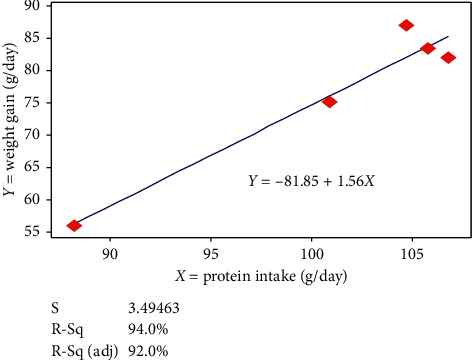
Linear regression relationship of daily body weight gain and protein intake of growing Bonga lambs fed on Rhodes grass hay basal diet and supplemented with dried mulberry and *Vernonia* mixed leaves' meal as a substitute to noug seed cake at different levels.

The functional dependence of daily weight again and daily protein intake of Bonga lambs fed on experimental diets is illustrated in Figure 3. Linear regression of daily protein intake versus daily weight gain was used to predict the weight performance of the animals.

## 4. Discussion

The high CP content of mulberry and *Vernonia* leaf meals indicates their high potential as a protein supplement diet for poor-quality roughage feed. The refusal hays contained lower crude protein and higher fibers (NDF, ADF, and ADL) as compared to offered hay indicating that more steamy and unpalatable part of refusals[24]. The lower crude protein and high fiber (NDF, ADF, and ADL) contents of the hay refusals than hay offered indicating occurrence of selection of the leaf part which are more palatable of the hay. The CP contents of noug seed cake (NSC) and ground maize grain in the present study were 32.4 and 8.3%, respectively. The CP content of NSC was comparable with 31.6% reported by Seid [25] and slightly lower than 33.3% reported by Dereje [26] and 33.4% by Gebru et al. [27]. The differences in the CP content of NSC may arise from variations in production management practices, agroecologies, soil conditions, and the efficiency of processing method employed [28].

The crude protein concentration of the dried mulberry leaf meal (DMLM) used in the present study was 18.5% and within the range of 15–30% reported by Boschini [28]. This CP value was higher than the values 15.2, 15.3, and 16.2% reported by Dereje [26], Yirga et al. [29], and Mitiku [30], respectively. The NDF content of dried mulberry leaves in the present study was 28.6% which is below 45% NDF grouped as high-quality feed [31]. The NDF content was lower than 32.9, 36.7, and 39.5% reported by Gebru et al. [27], Yirga et al. [29], and Dereje [26], respectively. The ADF content was within the range of 20.8–35% reported by Singh and Makkar [31]. The ADL content of dried mulberry leaves of the present study (7.4%) was higher than 4.6% reported by Mitiku [30] but lower than 11.8% and 11.1% reported by Dereje [26] and Yirga et al. [29], respectively. Generally, the difference in the nutritional composition of mulberry leaves might be due to variety, growing condition, phenological stage of the plant, age of the plant, and the leaf at harvest [31, 32]. The CP used in the present study was 22.5%. The high CP content of the dried *Vernonia* leaf meal (DVLM) indicates its high nutritive value and potential as a protein supplement to poor-quality roughages. This CP value is nearly similar with 22.6% reported by Woyessa et al. [33], but higher than 21.5 and 19.2% reported by Takele et al. [10] and Owen and Amakiri [34]. The current value of DM, ash, and ADL was comparable with 91.7, 4.7, and 5.06%, but the values of NDF and ADF were higher than the values reported by Woyessa et al. [33]. *Vernonia* leaves had higher NDF content (lower soluble carbohydrate content) than dried mulberry leaves.

The basal diet intake decreased with increasing the substitution levels of dried mulberry and *Vernonia* mixed leaves meal in T4 and T5 probably due to the bulkiness of the mixed leaves' meal contributing to the gut fills. Additionally, sheep supplemented with NSC mixtures showed greater basal intake than sheep fed with sole dried mulberry and *Vernonia* mixed leaves' meal which could be due to the high rate of digestion of diets containing NSC as compared to sole mixed leaves' meal [31].

Total dry matter intake (TDMI) and OM intake of sheep receiving T1, T2, T3, and T4 were significantly higher than T5. This might be due to that treatments containing NSC had reduced rumen retention time by increasing the outflow rate and stimulating the intake than sole mulberry and *Vernonia* mixed leaves' meal. Moreover, the physical and chemical characteristics of the feed can positively or negatively affect the intake [35]. The observed increase in DM and OM as a result of increasing the level of substitutions of mulberry and *Vernonia* mixed leaves' meal up to 75% in the present investigation agrees with the finding of Kedir [36] who noted greater DM and OM intakes of diets comprising *Vernonia* supplement in growing Somali goats fed with *Catha edulis* leftover. The total dry matter intake of sheep in the present study ranged 642.8–771.4 g/day and was higher than 480–498 g/day DM intake reported by Mulat [37] for local lambs fed on finger millet straw basal diet and different levels of concentrate supplements and comparable to 671.7–754.3 g/day reported by Teklu [38] for Arsi-Bale sheep fed on *Faba bean* straws with concentrate, but lower than 681.6–809.3 g/day reported by Animut and Adem [39] for Dorper x Afar F1 sheep fed on Rhodes grass hay supplemented with alfalfa, lablab, and *Leucaena leucocephala* and concentrate mixtures. The observed variations in dry matter intakes could be attributed to animal factor, body weight, environmental condition, diet composition, and quality [40].

The total dry matter intake (TDMI) in the present study ranged from 2.8 to 3.2% BW. The values were slightly higher than 2.8–3% reported by Teklu [38] for Arsi-Bale sheep fed on *Faba bean* straws with 300 g/day concentrate mix (noug seed cake and wheat bran) and lower than 3.25–4.29% BW reported by Yulistiani et al. [41]. Sheep which received T2, T3, and T4 statistically showed higher (*P* < 0.05) DM intake per unit of metabolic body weight (g/kgW^0.75^/day) than sheep in T5, but similar with T1. The variation in CP intake observed in the present study might be due to the differences in hay and supplement intakes. The total crude protein intake of the experimental animals in the present study ranged from 88.2 to 106.9g/day. The crude protein value lies between 81.1 and 121.6 g/day supplemented for Arsi Bale sheep breed fed on Faba bean haulms [42].

In the present study, the lower total NDF intake of lambs in T5 could be attributed to the lower total DM intake of lambs receiving T5 compared to the rest of the treatments. The ADF intake of lambs significantly varied (*P* < 0.01) among treatments. The highest ADF intake of lambs in T3 and the lowest in T5 might be due to high retention time of the undigested fiber content of feed in T5 which attributed to the lowest ADF intake. Statistically similar ADF intake was recorded between lambs in T2 and T4. The result of this study showed that increasing the substitution level of dried mulberry and *Vernonia* mixed leaves' meal for NSC up to 75% significantly increases the TDMI of the sheep as compared to the control group. However, the intake of the basal diet for sheep in T2, T3, and T4 was not significantly different with the intake in T1, but higher than T5.

The higher estimated metabolisable energy (EME) intake of sheep in T1, T2, T3, and T4 compared to sheep in T5 in this study could be associated with the higher content of NDF in T5 that resulted in lower metabolisable energy intake. The EME intake (8.3–10.2 MJ/day) in this experiment was higher than 6.2–8.5 MJ/day for the maintenance and growth requirements of sheep with 20–30 kg live weight [16]. This difference might be due to variations in breed, feed, and other factors.

Total dry matter intake increased with advancing feeding periods. This might be associated with increment in live body weight of experimental animals, which resulted in increased feed intake to satisfy nutrient requirement of the animal. The nutrient requirement of growing animals changes throughout the growing period in direct response to the changing needs of the individual organs and systems making up the whole animals [43]. The daily feed intake of sheep grouped in all treatments increased as the days of the feeding trial progressed.

The lower apparent DM and OM digestibility for sheep in T5 might be attributed to the higher NDF and ADF content in dried mulberry and *Vernonia* leaves as compared to NSC (see in Table 3). The result of the present study indicated that substitution of NSC protein with the dried mulberry and *Vernonia* leaf mixture up to 75% level (T4) did not affect the apparent digestibility of DM and OM. The higher CP digestibility with control (T1) and 75% level of NSC (T2) could be due to higher CP contents (see in Table 3) since high CP intake is usually associated with better DM digestibility [40]. The CP digestibility percent values observed in this study ranged from 73.9 to 81.8% which were higher than the digestibility values (68.2–71.9%, 73.8%) reported by Mitiku [30] and Tesfay et al. [44] for substitution of concentrate mix (noug seed cake and wheat bran) with dried mulberry leaf meal for Tigray lambs. The variation in CP digestibility might be due to the nutritional composition of the feed and animal type.

The level of apparent digestibility of diets in this study is an indication of improvement of digestibility of DM and nutrients of fibrous feed (hay) achieved due to supplements. McDonald et al. [40] supported that supplementation with forage legumes increased the digestibility of poor-quality roughages by promoting high microbial population by enhancing rumen fermentation process. Hence, based on the current study, dried mulberry and *Vernonia* mixed leaf meal was comparable to the concentrate mix in terms of diet digestibility and can be preferably used as a substitute to noug seed cake.

The final body weight of all experimental animals was increased during the feeding period for all treatments with a similar trend as total dry matter intakes. This result agrees with the report of Yulistiani et al. [41] who found that supplementation with mulberry as concentrate replacer had no significant effect on the final weight of Pelibuey male lambs. The differences in body weight change and daily body weight gain among treatments in the current study could be due to the differences in the amount of daily DM intake and digestibility of the feed intake [20]. Sheep which received T5 (100% DVLM and DMLM) had lower total body weight change (5.04 kg), and this value was slightly higher than 5.0 kg and lower than 6 kg as reported by Yirga et al. [29] that body weight change for local sheep fed with dried mulberry leaves at 1.5 and 2.5% body weight, respectively. Sheep fed with T5 had the lowest ADG, whereas sheep in T1 had the highest ADG. The variation in ADG might be resulted from sheep supplementation with NSC that had better DM and OM intake and nutrient digestibility than in T5. The level of intake and digestibility of experimental diets could determine animals' performances [35].

Furthermore, this difference might also be resulted from the relatively higher bypass protein content in NSC than the mulberry and *Vernonia* leaf mixture. Alemu and Merkel [6] in their study on evaluation of supplement feeds indicated that feeds such as oilseed cakes have large proportion of the protein bypasses to the small intestine without being solubilized in the reticulorumen, and this improves use of dietary protein more efficiently. The above authors also agreed that crude protein from forage legumes has intermediate rumen solubility. The values for ADG observed in the current study ranged 56.0–87.7 g/day and were higher than the values 51.9–70.7 g/day/head reported by Gebru et al. [27], who did substitution of concentrate mix with dried mulberry leaves in the diets of Abergelle sheep, and higher than 52–67 g/day for dried mulberry leaf supplementation for local sheep in Wollo area reported by Yirga et al. [29]. In contrary, the ADG observed in this study was lower than 93.8 g/day reported by Woyessa et al. [33] for Horro sheep supplemented with 450 g/day ground *Vernonia* leaves and ground sorghum grain mixtures. This result indicates that mulberry and *Vernonia* mixed leaves' meal can be replaced up to 75% NSC in sheep feeding strategy to mitigate the cost of feeding at smallholder farmers' level where mulberry and *Vernonia* trees are available. No differences were observed in feed conversion efficiency, indicating similarity in the age of the experimental animals, breed, and condition in which the animal was kept. This is in agreement with the finding of Woyessa et al. [33] for Horro sheep supplemented with different levels of ground *Vernonia* leaves and ground sorghum grain mixtures and Gebru et al. [27] for substitution of concentrate mix with dried mulberry leaves for Abergelle sheep. The present study has also showed no significant difference (*P* > 0.05) in protein conversion efficiency due to the treatment diets. The values for body weight change trends of T5 were lower compared to other treatments throughout the experimental period, which is consistent with the trend observed for dry matter intake. The regression graph tended to show that, for every one gram unit increase in protein intake, there was 1.56 g unit body weight gain of the experimental animal.

## 5. Conclusion and Recommendation

The current study indicates the substitutions of noug seed cake crude protein up to 75% dried mulberry and *Vernonia* mixed leaves' meal resulted in optimum performances in terms of feed intakes, digestibility of feeds, and growth performances of Bonga sheep. This study also highlights the positive potential of the dried mulberry and *Vernonia* leaves' mixture as a supplement to ruminants on a basal diet of fibrous feed. Therefore, considering the current availability, access, and cost of conventional concentrates and agroindustrial byproducts, using such browse trees in areas where they are available as an option of supplement regime in ruminant animal feeding is highly recommended. Verifying, demonstrating, and promoting the recommendation of this study to farmers are very important. Moreover, their potential year round availability of these fodder trees and their antinutritional content need additional study.

## Figures and Tables

**Table 1:** Proportion of different feed ingredients to formulate the experimental diets.

Treatments	Description	Supplement (g/day) DM basis						Tot supp. (g/day/h)
		RGH	GMG	NSC	DMLM	DVLM	Salt	
T1	100% concentrate mix	*Ad libitum*	198	198	—	—	4	400.0
T2	75% NSC + 25% (DVLM + DMLM)	*Ad libitum*	198	148.5	43.5	35.8	4	429.8
T3	50% NSC + 50% (DVLM + DMLM)	*Ad libitum*	198	99	87	71.5	4	459.5
T4	25% NSC + 75% (DVLM + DMLM)	*Ad libitum*	198	49.5	130.5	107.3	4	489.3
T5	100% (DVLM + DMLM)	*Ad libitum*	198	—	174	143	4	519.0

T = treatment; RGH = Rhodes grass hay; GMG = ground maize grain; NSC = noug seed cake; DVLM = dried *Vernonia* leaf meal; DMLM = dried mulberry leaf meal; g = gram; h = head (sheep).

**Table 2:** Chemical compositions of experimental feed ingredients, treatments diet, and offered and refused hay.

Sample description	Chemical compositions (%), DM						
	DM	OM	CP	Ash	NDF	ADF	ADL
Feed ingredients							
Hay offered	93.1	90.3	7.9	9.7	74.8	46.8	6.4
Mulberry leaf meal	92.1	83.3	18.5	16.7	28.6	26.6	7.4
*Vernonia* leaf meal	91.0	87.6	22.5	12.5	46.0	26.0	4.9
Ground maize grain	87.6	94.3	8.3	5.8	15.3	4.2	2.0
Noug seed cake	92.3	90.9	32.4	9.0	31.6	22.7	6.1
Treatments diets offered							
T1	89.9	94.2	19.8	5.8	23.5	13.5	3.6
T2	89.8	92.2	18.9	7.8	24.9	14.7	6.4
T3	89.9	90.6	17.9	9.4	26.2	15.9	5.6
T4	90.1	90.3	16.7	9.7	27.3	16.9	7.3
T5	90.0	89.5	15.8	10.5	28.3	17.8	8.1
Hay refusal							
T1	92.9	90.9	4.4	9.0	76.5	51.6	8.8
T2	92.8	91.5	4.5	8.5	78.4	53.2	8.9
T3	92.9	91.4	4.7	8.6	77.41	52.4	8.6
T4	92.7	90.7	4.3	9.3	77.5	52.4	8.9
T5	92.7	91.4	3.9	8.6	78.0	53.12	8.9

DM: dry matter; OM: organic matter; CP: crude protein; NDF: neutral detergent fiber; ADF: acid detergent fiber; ADL: acid detergent lignin; T1 = Rhodes grass hay *ad libitum *+ 100% concentrate mix; T2 = Rhodes grass hay *ad libitum* + 75% noug seed cake (NSC) and 25% dried mulberry and *Vernonia* mixed leaves' meal; T3 = Rhodes grass hay *ad libitum* + 50% NSC and 50% dried mulberry and *Vernonia* mixed leaves' meal; T4 = Rhodes grass hay *ad libitum* + 25% NSC and 75% dried mulberry and *Vernonia* mixed leaves' meal; T5 = Rhodes grass hay *ad libitum* + 100% dried mulberry and *Vernonia* mixed leaves' meal.

**Table 3:** Mean daily dry matter and nutrient intake of growing Bonga lambs fed on Rhodes grass hay basal diet and supplemented with dried mulberry and *Vernonia* mixed leaves' meal substituting noug seed cake of concentrate mix at different levels.

Intake (g/day)	Treatments					SEM	SL
	T1	T2	T3	T4	T5		
Hay DM	322.8^ab^	325.9^a^	323.3^ab^	294.1^b^	228.1^c^	9.41	^*∗∗*^
Supplement DM	400.0	429.8	448.1	465.6	414.7	—	—
TDMI	722.8^b^	755.7^ab^	771.4^a^	759.7^ab^	642.8^c^	13.34	^*∗∗*^
TDMI (%BW)	2.9	3.1	3.2	3.1	2.8	0.12	ns
TDMI (g/kgW^0.75^/day)	64.8^ab^	68.2^a^	70.6^a^	69.5^a^	60.9^b^	2.24	^*∗*^
Total nutrient intake							
OM	668.3^a^	689.1^a^	697.9^a^	686.1^a^	577.1^b^	12.06	^*∗∗*^
CP	104.7^ab^	106.8^a^	105.8^a^	100.9^b^	88.2^c^	1.48	^*∗∗∗*^
EME (MJ/kg DM)	10.0^a^	10.2^a^	9.9^a^	10.2^a^	8.3^b^	0.25	^*∗∗∗*^
Ash	54.5^c^	64.9^b^	73.5^a^	73.7^a^	65.7^b^	1.32	^*∗∗∗*^
NDF	335.5^a^	350.1^a^	359.3^a^	347.3^a^	288.1^b^	8.05	^*∗∗*^
ADF	204.9^b^	215.3^ab^	222.6^a^	216.4^ab^	180.6^c^	5.03	^*∗∗*^
ADL	35.1^c^	48.3^b^	45.8^b^	52.8^a^	48.2^b^	0.92	^*∗∗∗*^

^a,b,c^Mean values with different superscripts within a row are significantly different at ^*∗*^ = (*P* < 0.05), ^*∗∗*^ = (*P* < 0.01), and ^*∗∗∗*^ = (*P* < 0.001). DM: dry matter; OM: organic matter; CP: crude protein; EME: estimated metabolisable energy; NDF: neutral detergent fiber; ADF: acid detergent fiber; ADL: acid detergent lignin; TDMI: total dry matter intake; SEM: standard error of the mean; SL: significant level; ns: nonsignificant. T1 = Rhodes grass hay *ad libitum* + 100% concentrate mix; T2 = Rhodes grass hay *ad libitum* + 75% noug seed cake (NSC) and 25% dried mulberry and *Vernonia* mixed leaves' meal; T3 = Rhodes grass hay *ad libitum* + 50% NSC and 50% dried mulberry and *Vernonia* mixed leaves' meal; T4 = Rhodes grass hay *ad libitum* + 25% NSC and 75% dried mulberry and *Vernonia* mixed leaves' meal; T5 = Rhodes grass hay *ad libitum* + 100% dried mulberry and *Vernonia* mixed leaves' meal.

**Table 4:** Percentages of apparent DM and nutrient digestibility of experimental diets fed to growing Bonga lambs with Rhodes grass hay basal diet.

Digestibility (%)	Treatments					SEM	SL
	T1	T2	T3	T4	T5		
DM	70.8^a^	68.8^a^	69.1^a^	67.9^ab^	64.4^b^	1.25	^*∗*^
OM	73.8^a^	71.5^a^	70.94^a^	70.3^ab^	66.8^b^	1.22	^*∗∗*^
CP	81.8^a^	78.6^ab^	77.8^bc^	74.7^cd^	73.9^d^	1.16	^*∗∗∗*^
NDF	75.2^a^	63.8^b^	63.6^b^	62.7^b^	60.1^b^	1.55	^*∗∗∗*^
ADF	47.9	48.0	50.9	49.5	41.5	2.85	ns

^a,b,c,d^Mean values with different superscripts within a row are significantly different at ^*∗*^ = (*P* < 0.05), ^*∗∗*^ = (*P* < 0.01), and ^*∗∗∗*^ = (*P* < 0.001). DM: dry matter; OM: organic matter; CP: crude protein; NDF: neutral detergent fiber; ADF: acid detergent fiber; SEM: standard error of the mean; SL: significant level; ns: nonsignificant. T1 = Rhodes grass hay *ad libitum* + 100% concentrate mix; T2 = Rhodes grass hay *ad libitum* + 75% noug seed cake (NSC) and 25% dried mulberry and *Vernonia* mixed leaves' meal; T3 = Rhodes grass hay *ad libitum* + 50% NSC and 50% dried mulberry and *Vernonia* mixed leaves' meal; T4 = Rhodes grass hay *ad libitum* + 25% NSC and 75% dried mulberry and *Vernonia* mixed leaves' meal; T5 = Rhodes grass hay *ad libitum* + 100% dried mulberry and *Vernonia* mixed leaves' meal.

**Table 5:** Growth performances of Bonga lambs fed on Rhodes grass hay basal diet and supplemented with dried mulberry and *Vernonia* mixed leaves' meal substituting noug seed cake of the concentrate mix at different levels.

Parameters	Treatments					SEM	SL
	T1	T2	T3	T4	T5		
IBW (kg)	20.84	20.98	20.83	20.95	20.94	0.28	ns
FBW (kg)	28.74	28.36	28.34	27.72	25.98	0.67	ns
BWC (kg)	7.90^a^	7.38^a^	7.51^a^	6.77^a^	5.04^b^	0.49	^*∗∗*^
ADG (g/d/head)	87.7^a^	82.0^a^	83.4^a^	75.2^a^	56.0^b^	5.45	^*∗∗*^
FCE (ADG/daily TDMI)	0.12	0.11	0.11	0.09	0.08	0.001	ns
PCE (ADG/daily CPI)	0.79	0.73	0.75	0.69	0.58	0.05	ns

^a, b^Mean values within a row with different superscripts are significantly different at ^*∗∗*^ *=* (*P* < 0.01); SEM: standard error of the mean; SL: significant level; ns: nonsignificant; IBW: initial body weight; FBC: final body weight; BWC: body weight change; ADG: average daily gain; FCE: feed conversion efficiency; PCE: protein conversion efficiency. T1 = Rhodes grass hay *ad libitum *+ 100% concentrate mix; T2 = Rhodes grass hay *ad libitum* + 75% noug seed cake (NSC) and 25% dried mulberry and *Vernonia* mixed leaves' meal; T3 = Rhodes grass hay *ad libitum* + 50% NSC and 50% dried mulberry and *Vernonia* mixed leaves' meal; T4 = Rhodes grass hay *ad libitum* + 25% NSC and 75% dried mulberry and *Vernonia* mixed leaves' meal; T5 = Rhodes grass hay *ad libitum* + 100% dried mulberry and *Vernonia* mixed leaves' meal.

## Data Availability

The datasets used during the current study are available from the corresponding author on reasonable request.

## References

[1] Kumar S. N., Jayashankar M. R., Ramakrishnappa N., Ruban W., Sreesujatha R. M. (2017). Carcass and meat quality characteristics of Bandur ram lambs. *Indian Journal of Animal Research*.

[2] Abebe Y., Melaku S., Tegegne A. (2013). Assessment of sheep marketing system in burie district, north western Ethiopia. *Journal of Agricultural Research*.

[3] Federal Democratic Republic of Ethiopia Central Statistical Agency (CSA) (2017). Agricultural sample survey 2016/17. Volume II report on livestock and livestock characteristics private peasant holdings. *Statistical bulletin*.

[4] Gizaw S., Lemma S., Van Arendonk J. A. M., Komen H. (2007). Estimates of genetic parameters and genetic trends for live weight and fleece traits in Menz sheep. *Small Ruminant Research*.

[5] Edea Z. (2008). Characterization of Bonga and Horro indigenous sheep breeds of smallholders for designing community based breeding strategies in Ethiopia.

[6] Alemu Y., Merkel R. C. (2008). Nutrition and feeding of sheep and goats. *Sheep and Goat Production Hand Book*.

[7] Ensminger M. E. (2002). *Sheep and Goat Science*.

[8] Ebro S. G. A., Tesfaye Y., Mekuriaw Z. (2017). *Feed Resources in the Highlands of Ethiopia: A Value Chain Assessment and Intervention options*.

[9] Aynalem H., Taye T. (2008). The feed values of indigenous multipurpose trees for sheep in Ethiopia: the case of *Vernonia amygdalina, Buddleja polystachya* and *Maesa lanceolate*. *Livestock Research for Rural Development*.

[10] Takele G., Lisanework N., Getachew A. (2014). Evaluation of potential yield and chemical composition of selected indigenous multi-purpose fodder trees in three districts of wolayta zone, southern Ethiopia. *World Applied Sciences Journal*.

[11] Doran M. P., Laca E. A., Sainz R. D. (2007). Total tract and rumen digestibility of mulberry foliage (*Morus alba*), alfalfa hay and oat hay in sheep. *Animal Feed Science and Technology*.

[12] Valdes L. L. S., Borroto O. G., Perez G. F. (2017). Mulberry, Moringa and Tithonia in animal feed, and other uses. *Results in Latin America and the Caribbean*.

[13] Assen E., Getachew A., Mengistu U., Akililu (2015). Mulberry (*Morus alba*) as emerging potential opportunity for livestock feed development in northern Ethiopia. *Journal of Agricultural Science and Food Technology*.

[14] Ameha S., Zerihun T. (2011). *Design of an Experiment to Compare Performance and Meat Quality Characteristics of Three Ethiopian Goat Genotypes under Varying Nutritional Conditions*.

[15] Solomon G. S. A., Asfaw N. Growth response of horro sheep to different level of maize and noug seed cake supplements.

[16] ARC (Agricultural Research Council) (1980). The nutrient requirements of ruminant live stock. *Technical Review by an Agricultural Research Council Working Party Published on Behalf of the Agricultural Research Council by the Common Wealth Agricultural Bureau*.

[17] AFRC (Agricultural and Food Research Council) (1995). *Energy and protein requirements of ruminants. An advisory manual prepared by the AFRC Technical Committee on Response to Nutrients*.

[18] Getahun K. (2015). Optimum dietary crude protein level for fattening yearling arsi-bale lambs. *World Journal of Agricultural Sciences*.

[19] NRC (1996). *Nutrient Requirement of Sheep*.

[20] McDonald P., Edwards R. A., Greenhalgh J. F. D., Morgan C. A., Sinclair L. A., Wilkinson R. G. (2011). Nutrient requirements of the lactating ewe. *Animal Nutrition*.

[21] AOAC (1990). *Methods of Analysis*.

[22] Van Soest P. J., Robertson J. B. (1985). Analysis of forages and fibrous feeds. *Laboratory Manual for Animal Science*.

[23] SAS (2011). *Statistical Analysis System Version 9.3*.

[24] Van Soest P. J. (1994). *Nutritional Ecology of Ruminants*.

[25] Seid M. (2010). Feedlot performance, carcass and skin quality evaluation of arsi-bale goats and their 50% crosses with boer goats.

[26] Dereje W. (2015). Effect of substitution of concentrate mix with dried mulberry leaves on feed intake, digestibility, body weight gain and carcass characteristics of arsi-bale goats.

[27] Gebru D. T., Khushi Y. R., Tedla T. A. (2016). Substitution of dried mulberry (*Morus indica*) leaf meal for concentrate mix on feed intake, digestibility, and body weight gain and carcass characteristics of Abergelle sheep. *International Journal of Livestock Production*.

[28] Boschini C. (2006). Nutrientes digeribles, energía neta y fracciones proteicas de la morera (*Morus alba*) aprovechables en vacas lecheras. *Agronomía Mesoamericana*.

[29] Yirga M., Abreha S., Deribe B. (2016). The effect of mulberry leaf meal supplementation on feed intake and body weight change of indigenous Ethiopian highland sheep. *Abyssinia Journal of Science and Technology*.

[30] Mitiku A. (2011). Substituting concentrate mix by mulberry leaves (*Morus indica L*.) in the diet of lactating Holstein cows.

[31] Singh B., Makkar H. P. S., Sanchez (2002). The potential of mulberry foliage as a feed supplement in India. *Mulberry for Animal Production*.

[32] Sanchez M. D. (2002). *Mulberry: An Exceptional Forage Available Almost Worldwide. Mulberry for Animal production*.

[33] Woyessa F., Tolera A., Diba D. (2013). Feed intake, digestibility and growth performance of Horro lambs fed natural pasture hay supplemented graded level of *Vernonia* amygdalina leaves and sorghum grain mixture. *Science, Technology and Arts Research Journal*.

[34] Owen O. J., Amakiri A. O. (2011). Serological and haematological profile of broiler finishers fed graded levels of bitter leaf (*Vernonia amygdalina*) meal. *Advances Agriculture Biotechnology*.

[35] Guimarães G. S., Silva F. F. d., Silva L. L. d., Galvão L. M. G., Santos L. M. d., Alencar A. M. (2014). Intake, digestibility and performance of lambs fed with diets containing cassava peels. *Ciência e Agrotecnologia*.

[36] Kedir A. (2011). Effects of different levels of dried *Vernonia Amygdalina* leaf supplementation on feed intake, digestibility, weight gain and carcass parameters of somali goats fed *Catha edulis* leftover.

[37] Mulat A. (2006). Effects of Supplementing different protein source on feed intake and live weight gain of local sheep fed on finger millet (*Eleucine coracana*) straw basal diet.

[38] Teklu W. (2016). Effects of feeding different varieties of faba bean (*Vicia Faba* l.) straws with concentrate on feed intake, digestibility, and body weight gain and carcass characteristics of arsi-bale sheep.

[39] Animut G., Adem W. S. (2014). Digestibility and growth performance of dorper × afar f1 sheep fed rhodes grass (*Chloris Gayana*) hay supplemented with alfalfa (*Medicago Sativa*) lablab (*Lablab Purpures*) leucaena leucocephala and concentrate mixture.

[40] McDonald P., Edward RA., Green J. F. D., Morgan G. A. (2002). *Animal Nutrition*.

[41] Yulistiani D., Jelan Z. A., Liang J. B., Yaakub H., Abdullah N. (2014). Effects of supplementation of mulberry (*Morus alba*) foliage and urea-rice bran as fermentable energy and protein sources in sheep fed urea-treated rice straw based diet. *Asian-Australasian Journal of Animal Sciences*.

[42] Tesfaye N. (2008). Effect of supplementation with graded levels of wheat bran and “noug” seed cake mixtures on feed utilization of arsi-bale sheep fed urea treated maize cob basal diet.

[43] Pond K. R., Church C. D., Pond G. W. (1995). *Basic Animal Nutrition and Feeding*.

[44] Tesfay G., Tamir B., Berhane G. (2018). Substitution of mulberry leaf meal on feed intake, body weight and carcass characteristics of Tigray highland lambs. *Jurnal Ilmu Ternak Dan Veteriner*.

